# Comparative biomechanical analysis of volleyball court shoes during sport-specific tasks in competitive adolescent female volleyball players

**DOI:** 10.3389/fspor.2026.1824023

**Published:** 2026-05-12

**Authors:** Ava N. Davis, Ariel Huang, Ashley L. Erdman, Josh Riesenberg, Philip L. Wilson, Henry B. Ellis, Sophia Ulman

**Affiliations:** 1 Movement Science Lab, Scottish Rite for Children, Frisco, TX, United States; 2 Department of Orthopaedic Surgery, University of Texas Southwestern Medical Center, Dallas, TX, United States

**Keywords:** biomechanics, female athlete, footwear, motion analysis, volleyball

## Abstract

**Introduction:**

High-level volleyball requires efficient movement patterns, force attenuation, and rapid, multidirectional movements. While players may exhibit inherent biomechanical risk factors, investigating how footwear affects female athletes' movement is essential for developing a comprehensive understanding of determinants contributing to injury risk. The purpose of this study was to identify biomechanical differences associated with three volleyball court shoes.

**Methods:**

Twenty-nine healthy female volleyball players (15.52 ± 1.47 years, 168.72 ± 5.72 cm, 62.44 ± 7.71 kg) completed a countermovement jump (CMJ) and three-step approach in a motion capture laboratory using a markerless motion capture system and embedded force plates under three shoe conditions. Peak lower-extremity joint angles were calculated and extracted across the landing phase.

**Results:**

RIP-IT shoes elicited the least dorsiflexion and the greatest knee flexion and external rotation. Mizuno shoes were associated with greater contributors to dynamic knee valgus during the CMJ (*p* ≤ 0.013). Adidas shoes limited ankle inversion ROM compared to RIP-IT (*p* = 0.005) and ankle internal rotation compared to RIP-IT and Mizuno (*p* ≤ 0.015) during the CMJ.

**Discussion:**

Biomechanical risk factor associations differed across shoe conditions, with RIP-IT shoes limiting shock absorption strategies, Mizuno shoes increasing contributors to dynamic knee valgus, and Adidas shoes limiting ankle ROM. These results demonstrate that different volleyball shoes influence lower extremity kinematics and joint loading, with the most pronounced differences observed at the ankle and compensatory changes at the knee and hip, highlighting how footwear can measurably alter movement strategies during sport-specific tasks.

## Introduction

Volleyball is among the most popular sports worldwide, becoming one of the most-played sports at the high school level (1, 2). In the 2023–2024 school year, more than 564,000 high schoolers participated in the sport in the United States, with most players being female (2). With growing participation, volleyball-related injuries contribute to a significant portion of sport-related emergency department visits in the United States (3). To help manage and prevent injuries, many players have made intentional decisions about which volleyball shoes to wear, however, there is a lack of understanding on how footwear design (e.g., shape of shoe, heel-toe drop) influence mechanics and performance during dynamic sport tasks. Moreover, as more people play volleyball, options for volleyball shoes continue to grow, each brand marketing different advantages to optimize movement and appeal to players. This variability in options can make choosing a volleyball shoe difficult for youth athletes and their families. Shoes are an investment that can directly impact lower-extremity biomechanics. Therefore, it is important to make an informed decision that may reduce risky movement strategies (4, 5).

Volleyball players perform highly dynamic movements that require strength, power, and adaptability, such as jumping, landing, and multidirectional movements. These dynamic movements can lead to overuse injuries in the upper and lower extremities (6). Additionally, previous research has investigated differences in volleyball landing tasks, such that a single-leg landing, commonly found during the volleyball approach, was associated with high-risk movement strategies (7, 8). Between 2013 and 2022, lower extremity injuries, including non-contact anterior cruciate ligament (ACL) injuries, were the most prevalent among reported injuries associated with volleyball (3). In addition, lateral ankle sprains remain one of the most frequently reported injuries in volleyball and commonly occur through excessive ankle inversion during landing or rapid changes in direction (6, 9, 10). These statistics suggest the need for targeted prevention and injury risk reduction strategies. Previous volleyball shoe research has investigated specific factors, such as dorsiflexion range of motion (11), sole height and density (12), and shoe collar height (13). However, limited investigations have examined variability in lower extremity movement patterns and footwear design characteristics across sport-specific volleyball shoes, and how these parameters mechanically contribute to potential risk factors during repetitive loading.

This study aimed to evaluate whether different volleyball court shoes altered lower extremity biomechanics in adolescent female athletes performing sport-specific tasks. Identifying which type of volleyball court shoe promotes healthier loading strategies may impactfully contribute to reducing injury risk in adolescent female athletes. These findings could inform athletes, coaches, trainers, and parents about how footwear choices may produce differing biomechanical and performance characteristics during sport-specific tasks. Activities with highly dynamic movements, and therefore a greater risk of injury, make it especially important to identify strategies that reduce risk. It was hypothesized that lower extremity biomechanics would differ across the three volleyball shoes tested during sport-specific tasks. Investigating how footwear affects female athletes' movement is essential for developing a comprehensive understanding of all factors contributing to injury risk.

## Methods

### Study design and participants

**Figure 1:**
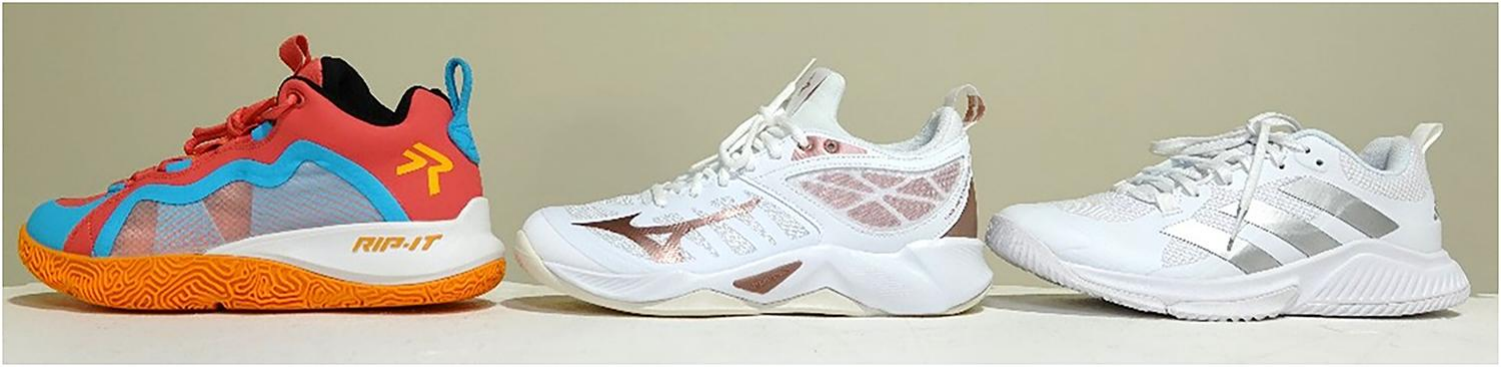
Volleyball shoe conditions. Left to right: RIP-IT SwiftStep FUTURE Volleyball Court Shoe, Mizuno Women's Wave Dimension Volleyball Shoe, Adidas Women's Court Team Bounce 2.0 Sneaker. All size 8.

The study followed a within-subjects, repeated-measures design to identify kinematic and kinetic differences between three volleyball court shoe conditions. A convenience sample of healthy adolescent female athletes who reported participating in volleyball competitively on a club/select team were tested. Participants were excluded if they reported a lower extremity injury six months prior to testing, or any neuromuscular or musculoskeletal conditions that would prevent their abilities to complete the jumping tasks. This study was approved by a regional Institutional Review Board and all participants provided informed written assent and parental consent prior to initiating testing procedures and for the publication of potentially identifiable images or data included in this article. For testing, all participants wore their preferred athletic attire and were tested under three different shoe conditions. The order in which participants wore the three shoe models was randomized and adequate rest periods were provided in between conditions to reduce the influence of fatigue on mechanics. The three volleyball court shoes tested in this study include the RIP-IT SwiftStep FUTURE Volleyball Court Shoe™, Mizuno® Women's Wave Dimension Volleyball Shoe, and Adidas® Women's Court Team Bounce 2.0 Sneaker, all of which are commercially available (Figure 1). To provide a relevant comparison, two commonly available volleyball shoes were selected alongside the female-specific RIP-IT shoes.

### Equipment and procedures

Kinematic data were collected using eight Qualisys Miqus cameras (Qualisys, Gothenburg, Sweden) and derived from markerless videos collected in 540p at 300 Hz, located on tripods approximately five feet from the ground. Kinetic data were computed using force data captured at 1,200 Hz from two embedded force plates (Advanced Mechanical Technology, Inc., Watertown, MA, USA). Kinematic and kinetic data were synced upon collection.

Prior to testing, participants donned all three pairs of shoes to ensure adequate fit. They were permitted to adjust sizes based on comfort and fit, which was confirmed by the research team. Notably, the size chosen was not always consistent across brands. Each participant's dominant leg was recorded by asking which leg the participant would kick a ball with and was used for subsequent analysis (14). All participants were right-leg dominant, and therefore this limb was used for kinematic and kinetic analysis. The dominant leg was used across tasks to ensure consistency. Additionally, each participant completed the Hospital for Special Surgery Pediatric Functional Activity Brief Scale (HSS Pedi-FABS). This validated questionnaire outputs a single score ranging from 0 to 30 with higher scores indicating greater activity levels and sport participation (15).

**Figure 2:**
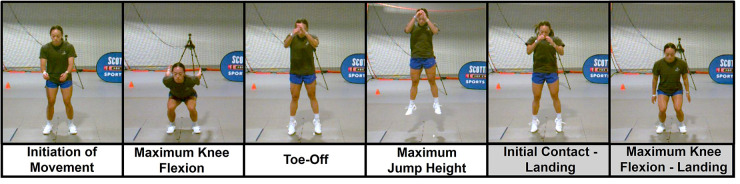
Countermovement jump (CMJ) task. Values for analysis were extracted between the shaded events, initial contact to maximum knee flexion of landing.

**Figure 3:**
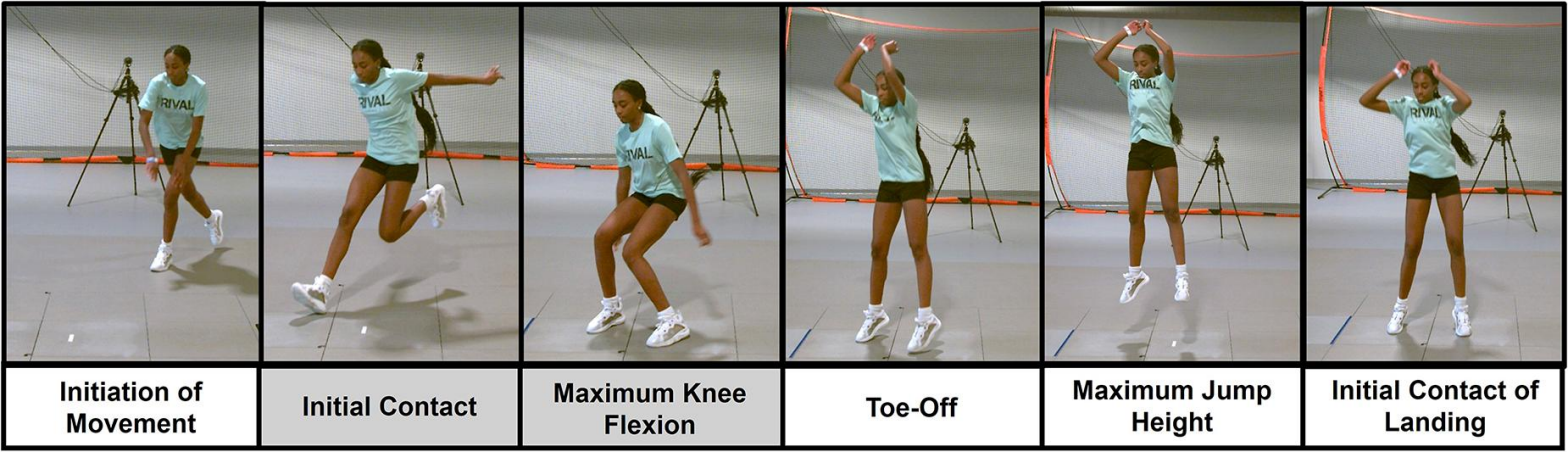
Three-Step approach task. Values for analysis were extracted between the shaded events, initial contact to maximum knee flexion prior to takeoff.

A warm-up period was required and performed prior to testing for each shoe condition to ensure familiarity with the shoes. The warm-up lasted an average of 3–5 min, depending on how comfortable the participant felt in the new shoes. Exercises included high knee skipping, bidirectional lateral shuffles, hamstring activation jog, and countermovement jumps. Following the warm-up, a static trial was captured. Participants then completed three trials of a countermovement jump (Figure 2) and three trials of a three-step volleyball approach (Figure 3). These procedures were repeated for each shoe condition, and the order of shoe condition was randomized for each participant.

### Countermovement jump (CMJ)

For the CMJ, participants were instructed to start with one foot in each force plate and feet shoulder-width apart. Then, by first swinging their arms down (countermovement), they were asked to jump up as high as they could before landing back with one foot in each of the two force plates. Trials were excluded if the task was completed incorrectly (e.g., tuck position, minimal effort) or if either foot landed out of the force plates. Trials were completed until the participant successfully completed three sufficient trials. All kinematic and kinetic measures were extracted from the landing phase of the task, defined as initial contact to maximum knee flexion (Figure 2). Initial contact was defined using a vertical ground reaction force threshold of >20N.

Verbal Instructions: “Please start by standing with one foot in each square [force plate] with your feet shoulder-width apart. When I say go, use your arms to jump up as high as you can and land back in the starting position, with each foot in a square.”

### Three-step approach

The three-step approach is a fundamental technique used primarily by front-row volleyball players to generate significant momentum and power for a vertical jump to hit the ball over the net. While there are variations to the approach (four-step, three-step, etc.), the common purpose is to optimize jump height, timing, and power. This movement allows players to convert forward momentum into a vertical jump, which is important for having a successful hit to the opponent. The first step is short and quick, initiating forward movement. The second step is longer and faster, accelerating the body, and is most important for transferring horizontal velocity into upward force. The third step functions as the plant and breaking phase, during which forward motion is reduced, center of mass is lowered, and the body is prepared for vertical propulsion (16).

For the three-step volleyball approach task, participants had the option of approaching from the left side, right side, or middle, depending on their primary position and which felt most natural. After choosing their approach angle, participants were instructed to complete the same angle for each shoe condition to ensure the within-subject comparison remained consistent. The final two steps prior to jumping were to be completed with one foot in each force plate. Participants' dominant limb, which would be the first limb planted in the force plate during the three-step approach, was used for analysis. Participants were also instructed that they were not required to land back in the force plates as this task typically involves a slight jump forward in a game or practice environment. Trials were excluded if foot placement at initial contact was not in each individual force plate at initial contact. The loading phase for the three-step approach was defined as initial contact to maximum knee flexion prior to takeoff (Figure 3).

Verbal Instructions: “When I say go, please perform the three-step appriach, making sure your final two steps before jumping up are in the two squares [force plates]. Try to keep one foot ine ach square before takeoff, and then jump up as high as you can as if you were hitting at the net. You are not required to land back in the two squares. Feel free to land how you normally would after hitting at the net.”

### Data processing and analysis

Kinematic data were processed through Theia3D markerless motion capture software (version 2023.2.0.3160, Theia3D, Theia Markerless Inc., Kingston, ON, Canada) and filtered using an integrated 20 Hz spline filter. Kinetic data were filtered using a 4th order low-pass Butterworth filter at 16 Hz (17). Theia3D utilizes a machine learning model to identify 124 anatomical landmarks on the body in each frame of collected video. The calibrated cameras allow Theia to accurately and reliably reconstruct 3D pose estimations of the body throughout the movement (18, 19). The final Theia pose outputs were sent to Visual3D (version 2024.06.1, HAS-Motion Inc., Canada) where peak and range-of-motion (ROM) joint and segment angles and (external) moments in the sagittal, coronal, and transverse planes were calculated using standard model formulas, creating a full body model of motion from inverse kinematics. Given the high incidence of ankle injuries in volleyball and the potential influence on frontal-plane ankle stability, ROM was specifically computed for ankle inversion/eversion to better quantify the amount of motion permitted in each shoe condition. This variable was extracted because lateral ankle sprains, which commonly occur through excessive ankle inversion, are prevalent in volleyball, and evaluating whether footwear influences ankle frontal-plane ROM may provide insight into potential injury-related mechanics. For subsequent analysis, individual kinematic and kinetic measures were averaged across the three trials for each task to better represent each participant's typical movement strategy.

### Statistical analysis

Descriptive statistics were performed to capture means and standard deviations for all kinematic and kinetic variables. Given significant Shapiro–Wilk tests of normality, non-parametric analyses were conducted. Specifically, following the within-subjects, repeated measures design, Friedman tests were performed to assess the effects of shoe condition on outcome measures of the dominant limb during the CMJ and three-step approach tasks. For significant Friedman tests, Wilcoxon signed-rank tests were performed for *post hoc* paired comparisons, with effect size (*r*) calculated to assess the magnitude of this difference and interpreted as small (0.10), moderate (0.30), or large (≥ 0.50) (20). Statistical significance level (*α*) was set to 0.05, and Bonferroni corrections were applied for *post hoc* paired comparisons (i.e., *α* = 0.05/3 comparisons=0.017). All statistical tests were evaluated in SPSS Statistics (IBM, version 24.0, Armonk, NY, USA).

## Results

Twenty-nine healthy female volleyball players (15.52 ± 1.47 years, 168.72 ± 5.72 cm, 62.44 ± 7.71 kg) actively competing on a club team were tested from June to July 2025. Among the 29 athletes tested, the largest position groups were Outside Hitters (*n* = 9) and Opposite Hitters (*n* = 9), followed by Setters (*n* = 7), Defensive Specialists (*n* = 2), Middle Blocker (*n* = 1), and Libero (*n* = 1). Participants scored an average of 21.90 ± 4.57 out of 30 on the Pedi-FABS, indicating a relatively high activity level. The mean shoe size selected differed across models with RIP-IT (9.82 ± 1.22, 7.5–11.5) shoe sizes significantly larger than both Mizuno (9.32 ± 1.10, 7.5–11), and Adidas (9.18 ± 1.21, 7–11; *p* < 0.001 each). Average (SD) as well as Wilcoxon signed-rank test results across shoe conditions for the CMJ and the three-step approach are presented in Tables 1, 2, respectively. There were no significant differences in peak ground reaction force (GRF) or jump height for the CMJ or three-step approach.

**Table 1:** Kinematic and kinetic measures between RIP-IT, Mizuno, and Adidas volleyball court shoes and Wilcoxon signed-rank test results for the CMJ.

Countermovement Jump	RIP-IT	Mizuno	Adidas
Kinematics (°)	Mean ± SD	Mean ± SD	Mean ± SD
Ankle			
Dorsiflexion	25.71 ± 3.69	28.21 ± 4.45^R^	26.88 ± 4.50^M^
Inversion	10.61 ± 2.65	10.72 ± 2.06	11.37 ± 2.76
Inversion ROM	5.93 ± 2.60	5.25 ± 2.12	4.71 ± 1.66^R^
Internal Rotation	9.57 ± 3.74	7.53 ± 3.55^R^	6.58 ± 3.05^R,M^
Knee			
Flexion	79.11 ± 14.19	75.76 ± 14.10^R^	75.63 ± 13.84^R^
Valgus	6.16 ± 2.05	6.52 ± 2.82	5.79 ± 2.50
External Rotation	13.48 ± 3.13	11.86 ± 3.29^R^	12.59 ± 3.06^R^
Hip			
Flexion	56.44 ± 18.02	53.71 ± 17.05^R^	53.35 ± 18.23
Abduction	7.15 ± 13.21	7.39 ± 3.12	7.51 ± 13.54
Internal Rotation	3.10 ± 2.47	2.52 ± 1.99	2.14 ± 2.55^R^
Kinetics (Nm/kg)			
Ankle			
Dorsiflexion Moment	1.32 ± 0.31	1.34 ± 0.27	1.33 ± 0.30
Inversion Moment	0.20 ± 0.10	0.16 ± 0.10^R^	0.15 ± 0.08^R^
Internal Rotation Moment	0.09 ± 0.07	0.09 ± 0.06	0.08 ± 0.06
Knee			
Flexion Moment	1.66 ± 0.35	1.64 ± 0.42	1.60 ± 0.38
Valgus Moment	0.20 ± 0.08	0.20 ± 0.11	0.19 ± 0.11
External Rotation Moment	0.16 ± 0.09	0.17 ± 0.08	0.17 ± 0.11
Hip			
Flexion Moment	1.07 ± 0.39	1.06 ± 0.33	1.05 ± 0.41
Adduction Moment	0.60 ± 0.26	0.64 ± 0.25	0.61 ± 0.25
Internal Rotation Moment	0.41 ± 0.13	0.41 ± 0.13	0.40 ± 0.13
Ground Reaction Force			
Vertical	1.65 ± 0.41	1.68 ± 0.44	1.65 ± 0.37
Posterior	0.15 ± 0.08	0.15 ± 0.10	0.14 ± 0.08
Jump Height (cm)	39.07 ± 6.40	38.80 ± 5.96	38.63 ± 6.28

Superscripts indicate statistically significant differences between shoe conditions. A value marked with ^R^ indicates a significant difference with RIP-IT and ^M^ indicates a significant difference with Mizuno during the landing phase of the CMJ.

**Table 2:** Kinematic and kinetic measures between RIP-IT, Mizuno, and Adidas volleyball court shoes and Wilcoxon signed-rank test results for the three-step approach.

Three-Step Approach	RIP-IT	Mizuno	Adidas
Kinematics (°)	Mean ± SD	Mean ± SD	Mean ± SD
Ankle			
Dorsiflexion	14.71 ± 5.11	18.29 ± 5.60^R^	17.03 ± 4.75^R^
Inversion	6.05 ± 4.01	8.15 ± 4.11^R^	7.27 ± 4.48
Inversion ROM	3.42 ± 3.07	3.56 ± 2.81	3.21 ± 2.78
Internal Rotation	9.20 ± 5.02	7.81 ± 5.34	8.41 ± 5.02
Knee			
Flexion	80.94 ± 9.31	80.86 ± 8.72	80.54 ± 9.07
Valgus	6.06 ± 3.35	7.54 ± 3.77^R^	6.05 ± 3.51^M^
External Rotation	12.77 ± 4.23	11.31 ± 3.80	11.73 ± 3.52
Hip			
Flexion	66.78 ± 6.46	65.35 ± 7.03	66.72 ± 7.32
Adduction	3.73 ± 4.53	3.63 ± 4.39	2.66 ± 4.07
External Rotation	2.24 ± 3.43	2.02 ± 3.10	2.34 ± 3.35
Kinetics (Nm/kg)			
Ankle			
Dorsiflexion Moment	1.04 ± 0.29	1.15 ± 0.29^R^	1.12 ± 0.31
Inversion Moment	0.19 ± 0.10	0.18 ± 0.11	0.19 ± 0.11
Internal Rotation Moment	0.16 ± 0.12	0.15 ± 0.14	0.16 ± 0.14
Knee			
Flexion Moment	1.93 ± 0.40	1.92 ± 0.40	1.92 ± 0.36
Valgus Moment	−0.05 ± 0.20	0.02 ± 0.25^R^	−0.04 ± 0.23
External Rotation Moment	0.34 ± 0.15	0.30 ± 0.16	0.29 ± 0.15
Hip			
Flexion Moment	1.78 ± 0.42	1.74 ± 0.39	1.77 ± 0.41
Adduction Moment	0.74 ± 0.29	0.75 ± 0.32	0.69 ± 0.31
Internal Rotation Moment	0.69 ± 0.19	0.73 ± 0.15	0.72 ± 0.20
Ground Reaction Force			
Vertical	1.33 ± 0.32	1.35 ± 0.21	1.35 ± 0.19
Posterior	0.32 ± 0.11	0.33 ± 0.13	0.34 ± 0.12
Jump Height (cm)	41.67 ± 7.18	42.32 ± 6.24	41.66 ± 6.75

Superscripts indicate statistically significant differences between shoe conditions. A value marked with ^R^ indicates a significant difference with RIP-IT and ^M^ indicates a significant difference with Mizuno during the three-step approach task.

### Countermovement jump (CMJ)

#### Ankle

RIP-IT shoes allowed the least amount of dorsiflexion compared to both Mizuno and Adidas, but only significantly different from Mizuno (*p* < 0.001, *r* = 0.66). Mizuno allowed the greatest amount of dorsiflexion and differed significantly with Adidas (*p* = 0.009, *r* = 0.48), indicating a moderate effect size. While there were no significant differences in peak ankle inversion, there were significant findings in overall inversion ROM during the landing phase. RIP-IT allowed for the greatest range-of-motion across all conditions and differed significantly with Adidas (*p* = 0.005, *r* = 0.52). Additionally, ankle internal rotation differed across all three conditions. RIP-IT resulted in the greatest internal rotation and was significantly different than Mizuno (*p* = 0.001, *r* = 0.65) and Adidas (*p* < 0.001, *r* = 0.77), indicating large effect sizes. Similarly, Mizuno had significantly greater ankle internal rotation during landing compared to Adidas (*p* = 0.015, *r* = 0.45). Lastly, RIP-IT elicited a significantly greater ankle inversion moment compared to Mizuno (*p* = 0.001, *r* = 0.59) and Adidas (*p* < 0.001, *r* = 0.74), indicating large effect sizes.

### Knee

RIP-IT shoes elicited greater knee flexion compared to both Mizuno (*p* < 0.001, *r* = 0.66) and Adidas (*p* = 0.003, *r* = 0.55) during the CMJ landing phase. Additionally, RIP-IT shoes resulted in the greatest external knee rotation compared to Mizuno (*p* < 0.001, *r* = 0.65) and Adidas (*p* = 0.002, *r* = 0.56), indicating large effect sizes, suggesting the magnitude of this difference was high. There were no significant differences in knee valgus or knee moments among shoe conditions.

### Hip

During landing, the RIP-IT shoes were associated with the greatest hip flexion and differed significantly with Mizuno (*p* = 0.017, *r* = 0.44). When wearing the RIP-IT shoes, hip internal rotation was greatest and differed significantly compared to the Adidas shoes (*p* = 0.003, *r* = 0.56), indicating a relatively large effect size. No differences between shoe conditions were observed for hip moments.

### Three-step approach

#### Ankle

Across all three shoe conditions, RIP-IT elicited significantly less dorsiflexion compared to both Mizuno (*p* < 0.001, *r* = 0.68) and Adidas (*p* = 0.005, *r* = 0.52). Additionally, RIP-IT displayed significantly less inversion than Mizuno (*p* < 0.001, *r* = 0.66), indicating a large effect size. The only kinetic difference at the ankle was RIP-IT displayed significantly less dorsiflexion moment compared to Mizuno (*p* = 0.009, *r* = 0.49).

#### Knee

Landing with Mizuno elicited a significantly greater amount of knee valgus compared to both RIP-IT (*p* = 0.001, *r* = 0.60) and Adidas (*p* = 0.013, *r* = 0.46), suggesting a moderate effect size. Mizuno was the only shoe condition resulting in a valgus moment, whereas RIP-IT differed significantly with a varus moment (*p* = 0.007, *r* = 0.50), demonstrating the magnitude of this difference was moderate. There were no differences in sagittal or transverse plane knee kinematics or kinetics between shoe conditions.

#### Hip

There were no significant kinematic or kinetic findings at the hip across the three shoe conditions for the three-step approach.

## Discussion

The present study examined how three sport-specific volleyball shoes influence lower extremity biomechanics during common volleyball tasks. The findings indicate that footwear condition was associated with distinct movement strategies across the ankle, knee, and hip joints, suggesting that structural features of volleyball shoes may influence how players dissipate impact upon landing. Interestingly, the RIP-IT shoes consistently demonstrated limited ankle dorsiflexion, as well as increased frontal and transverse plane motion at the knee during the CMJ landing, while Mizuno shoes were associated with greater knee valgus during the three-step approach. Greater variability in both hip and knee joint angles across tasks further highlights how subtle shoe design parameters can influence individual movement strategies. These results emphasize the potential role of footwear selection in influencing biomechanical performance and injury risk factors during sport-specific tasks.

### Ankle

Across the CMJ and three-step approach tasks, the RIP-IT shoes consistently reduced ankle dorsiflexion compared to Mizuno and Adidas. Previous research has investigated dorsiflexion ROM, and its effects on landing mechanics (21, 22). Limited dorsiflexion has previously been associated with compensatory movement strategies, such as increased hip and knee flexion, which may alter force absorption patterns, potentially increasing risk of injury (23, 24). Therefore, the reduction of ankle motion in the sagittal plane in the RIP-IT shoes may impact joint loading patterns during dynamic tasks, creating stiffer landing strategies at the ankle. This further explains why RIP-IT shoes elicited the most knee and hip flexion, possibly compensating for the lack of force absorption down the kinetic chain.

Notably, during the CMJ, RIP-IT shoes also allowed for the greatest ankle inversion ROM and internal rotation upon landing, indicating increased movement excursion rather than higher peak inversion. These two components are common mechanisms in lateral ankle sprains (25, 26). However, because the relationship between ankle inversion ROM and injury risk remains unclear, these differences in ankle motion, both in the RIP-IT shoe and the other sport-specific models, warrants further investigation during high-impact landings. These findings may be attributed to aspects of the RIP-IT shoe's design, which may encourage a slightly more everted foot orientation. The greater ankle medial-lateral ROM in the RIP-IT shoe may reflect variable lateral stability or medial support during use. RIP-IT shoes are designed specifically for the female foot, having a wider toe-box, lower volume instep, and narrower heel (27). Having more space in the toe-box (common in female-specific shoes) can allow for greater ankle ROM, effectively dissipating impact forces, distributing load, and facilitating balance during landings (28, 29). However, excessive ROM in the ankle can lead to instability upon landing (30). Differences in ankle mechanics across footwear conditions may partially be explained by structural features such as heel-toe drop, collar height, and outsole design, all of which can influence frontal plane stability and sagittal plane motion during landing. Future work should more directly investigate the influence of shoe structural properties on movement strategies and how different components of footwear affect stability and loading patterns.

### Knee

During the CMJ landing, RIP-IT shoes elicited significantly greater knee flexion compared to Adidas and Mizuno. Greater knee flexion during landing is often interpreted as a more efficient shock-absorption strategy, distributing ground reaction forces evenly up the kinetic chain (23, 24). This suggests that the RIP-IT shoes may facilitate a “softer” landing strategy compared to the Mizuno and Adidas volleyball court shoe. However, previous research has shown that an increase knee flexion angle is associated with an increased dorsiflexion angle (21, 31). Alternatively, the current study showed the greatest knee flexion in RIP-IT shoes, but the least ankle dorsiflexion upon CMJ landing. This leads to the possibility that greater knee flexion in the RIP-IT shoe condition was elicited as a compensatory response to limited ankle dorsiflexion to absorb impact forces (32). Although not statistically significant during the three-step approach, the RIP-IT shoe condition still averaged the greatest knee flexion, supporting a consistent trend across tasks.

In addition, the RIP-IT shoes facilitated the greatest knee external rotation during CMJ landing compared to Mizuno and Adidas shoes. Excessive knee external rotation has been linked to increased strain on the medial knee structures, potentially predisposing athletes to overuse injuries (33). Moreover, excessive tibial (knee) rotation can contribute to dynamic knee valgus during landing, a common biomechanical risk factor for ACL injury, especially during complex movements (34–37). Previous research has found that reduced ankle dorsiflexion ROM upon landing is associated with greater knee external rotation angles (21, 38). Interestingly, in the current study, Mizuno shoes were associated with significantly greater knee valgus than both Adidas and RIP-IT shoes during the three-step approach, suggesting that the Mizuno shoes may compromise frontal plane knee stability and control the most out of the three shoe conditions (39). Likewise, these findings suggest that both lower-limb mechanics and footwear choice may interact to influence dynamic knee valgus during multiplanar movements like the three-step approach, which has been previously associated with mechanisms linked to ACL injury in landing tasks (37).

### Hip

At the hip, there were distinct biomechanical differences between the three shoe conditions. During the CMJ, participants exhibited greater hip flexion wearing the RIP-IT shoes than both Mizuno and Adidas, suggesting improved shock absorption strategies during landing (40, 41). However, since RIP-IT shoes limited dorsiflexion, greater hip flexion in addition to greater knee flexion may be another compensatory strategy to dissipate forces up the kinetic chain and maintain energy attenuation (42). Furthermore, RIP-IT shoes elicited the greatest hip internal rotation during the CMJ, differing significantly with Adidas. While moderate hip internal rotation is typical during landing, especially in females, excessive internal rotation can be problematic when accompanied by other biomechanical risk factors such as knee valgus and hip adduction (43, 44). Research has shown that individuals with greater hip internal rotation ROM exhibit increased medial knee displacement, a strong component of dynamic knee valgus, during landing tasks (45, 46). While current literature does not specifically define a universally accepted numeric threshold as “excessive” for hip internal rotation, it is found that combined with hip adduction, knee valgus, and external tibial rotation, greater than 10° is a biomechanical risk factor (47). In the current study, the relatively elevated hip internal rotation with the RIP-IT footwear may therefore warrant caution when combined with other contributors to dynamic knee valgus.

### Influence of shoe condition on biomechanics associated with injury risk

While injury outcomes were not directly measured in this study, the observed biomechanical differences across footwear conditions may provide insight into movement strategies associated with common lower extremity injury mechanisms in volleyball. Lateral ankle sprains frequently occur through excessive ankle inversion and internal rotation during landing tasks, while dynamic knee valgus and knee external rotation have been associated with mechanisms underlying non-contact ACL injuries (6, 37). Previous studies have highlighted risks associated with staggered landing, commonly seen following the three-step approach (7, 8), that may become even more of a risk with footwear that elicits improper landing mechanics. In the current study, differences in ankle inversion ROM, knee valgus, and transverse plane joint rotations suggest that footwear design may influence lower extremity movement strategies during volleyball-specific tasks. However, these findings should be interpreted with caution, as the presence of biomechanical differences does not directly indicate increased injury risk. Rather, the results highlight how footwear characteristics may influence movement patterns previously associated with injury risk during high impact landings.

### Limitations

The current study has a few limitations that should be noted. Given the current study was conducted on healthy, adolescent female volleyball players, the results presented here may not be generalizable to older female or male populations. Future work should expand the scope of the population to investigate if different sport-specific footwear elicits variable biomechanics in male and female athletes of different sports. Additionally, the sample size (*n* = 29) was modest given the variables and conditions tested. Future studies should include a greater sample size to allow an opportunity for improved reliability and generalizability of the findings. Additionally, the current study was conducted over a limited period, which may not reflect how the shoes perform with long-term use (i.e., durability, wear, adaptation). Testing was conducted in a controlled environment (motion capture laboratory) rather than a game-like environment where tasks are highly variable and dependent on team performance. The three-step approach area of interest was the plant phase prior to jumping due to the ability to retrieve kinetic data. Future work should additionally focus on the landing phase after the approach to get a well-rounded analysis and comparison across different shoes. Additionally, the present study did not directly quantify structural properties of the footwear (e.g., collar height, toe box width), which limits the ability to determine which specific design features contributed to the observed biomechanical differences. Following the completion of data collection, all shoes were returned and therefore additional measurements and images could not be obtained. As a result, specific footwear characteristics that may have influenced the observed biomechanical differences between conditions could not be directly evaluated. Future work should investigate specific structural design parameters in sport-specific footwear to determine the influence it has on movement strategies.

While some of the statistically significant differences observed in this study were relatively small in magnitude (e.g., < 2°), these findings remain biomechanically meaningful. In high-level sports, subtle deviations in joint kinematics or kinetics may influence cumulative loading strategies, but the precision of markerless motion capture during fast, multiplanar movements can introduce measurement error that approaches the magnitude of these differences. Nonetheless, consistent directional trends were observed across multiple joints and tasks, suggesting the concept that footwear design can influence movement strategies. The statistically significant findings, despite small numerical differences, highlight sensitivity and reliability of motion capture and support the presence of systematic variation across the three shoe conditions.

## Conclusion

This study highlights that sport-specific footwear may significantly impact lower extremity kinematics and kinetics during volleyball-specific tasks such as the CMJ and three-step approach. Differences were most pronounced within the sagittal and rotational planes of the ankle, with compensatory changes observed at the knee and subtle adaptations at the hip. Notably, the RIP-IT shoe consistently limited dorsiflexion, and increased knee external rotation, while the Mizuno shoe was associated with movement patterns contributing to dynamic knee valgus. These findings suggest that athletes subtly adapt their movement strategies in response to different footwear constraints, though the direct practical impact on performance or injury risk remains uncertain. Athletes, clinicians, and coaches may consider these biomechanical trends when selecting shoes for sport-specific tasks, specifically those that challenge ankle, knee, and hip mechanics to promote efficient movement strategies.

## Data Availability

The raw data supporting the conclusions of this article will be made available by the authors, without undue reservation.
